# Supplementary figures and images for: Virus-like particle-mediated delivery of structure-selected neoantigens demonstrates immunogenicity and antitumoral activity in mice
Source: J Transl Med. 2024 Jan 3;22:14. doi: 10.1186/s12967-023-04843-8 (PMC10763263; doi:10.1186/s12967-023-04843-8)

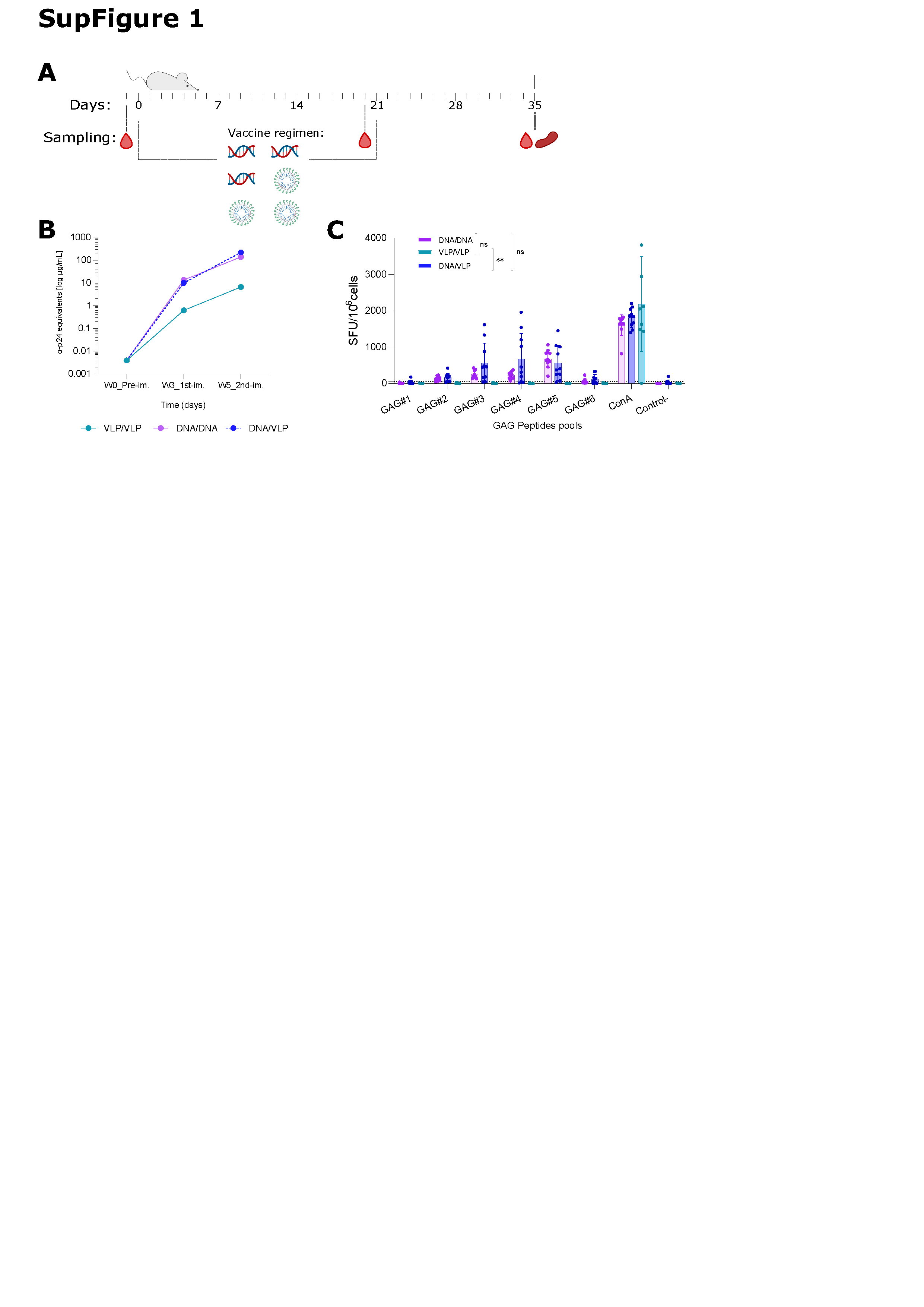

Supplement: Supplementary file 2 — Additional file 2: Figure S1. Selecting the vaccination regimen for the highest immune response. (A) Experimental design for testing neoVLP vaccine regimen. (B) Evaluation of the humoral response generated against recombinant Gag at sacrifice. DNA/DNA regimen in purple, DNA/VLP regimen in blue and VLP/VLP regimen in turquoise. (C) Evaluation of T cell response against pools of peptides covering the HIV-1 Gag protein. DNA/DNA regimen in purple, DNA/VLP regimen in blue and VLP/VLP regimen in turquoise. [file 12967_2023_4843_MOESM2_ESM.tiff]

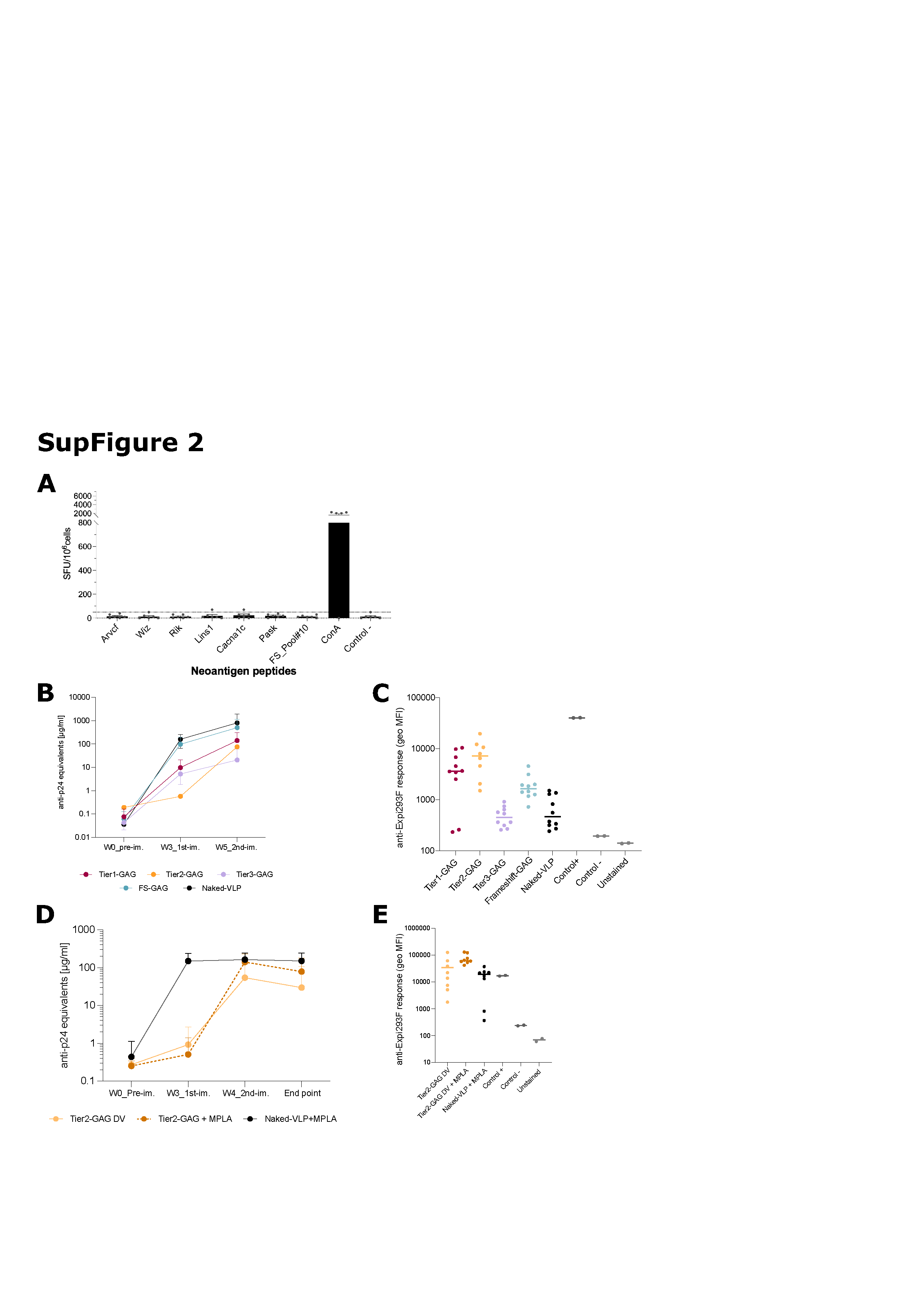

Supplement: Supplementary file 3 — Additional file 3: Figure S2. Natural tumor immunogenicity against neoantigens and humoral response against Gag and host cell proteins. (A) Cellular response generated by natural tumor immunogenicity against selected neoantigens. (B) Evaluation of humoral response against HIV-1 Gag over time for all groups vaccinated with neoVLPs. (C) Evaluation of the humoral response against Expi293F proteins at each endpoint for all groups vaccinated with neoVLPs. Level of response in vaccinated animals is displayed as coloured dots according to each group. Staining controls are shown as grey dots. (D) Evaluation of humoral response against HIV-1 Gag for groups vaccinated with Tier2-GAG (in yellow), Tier2-GAG + MPLA (in brown) and naked-VLP + MPLA. (E) Evaluation of the humoral response against host proteins at endpoint for groups vaccinated with Tier2-GAG (in yellow), Tier2-GAG + MPLA (in brown) and naked-VLP + MPLA (in grey). [file 12967_2023_4843_MOESM3_ESM.tiff]
